# Mr. Mills, on a Case of Uterine Hydatids

**Published:** 1799-12

**Authors:** S. Gillam Mills

**Affiliations:** Croom's Hill


### *To the Editors of the Medical and Phyſical Journal.*

Gentlemen,

As there are not many caſes on record, ſimilar to the one deſcribed in the annexed paper, you poſſibly may think it worthy a place in your valuable Publication.

I am, Gentlemen,

With great reſpect,

Your moſt obedient humble ſervant,

*Croom's Hill, Oct.* 12, 1799. S. GILLAM MILLS.

SOME time in July 1797, I was caſually conſulted by a lady, who imagined herſelf two or three months gone with child; her complaints were were pains in the back, attended at times with the ſenſe of bearing down, and appearances of catamenia; hence ſhe imagined ſhe was about miſcarrying. Knowing her averſion to any medical regimen, I only adviſed her to keep as much as poſſible in an horizontal poſition, and to pay due attention to the ſtate of her bowels. Several weeks after I had given this advice, I met her maid, who informed me, that her miſtreſs continued much as when I ſaw her; from this, I hinted as my opinion, that ſhe was not pregnant.

When my patient had ſuppoſed herſelf to be five or ſix months gone, I was deſired (unknown to her) to viſit her; ſhe was then on the bed, and told me ſhe had had throughout the day regular pains, with ſome diſcharge; but as ſhe thought the pains were not ſufficient to require my aſſiſtance, ſhe had neither ſent to me or the nurſe, being ſtrongly averſe to having either with her till abſolutely neceſſary. I deſired the family would immediately ſend for the nurſe, to whom I gave directions that were proper, for obtaining a due information reſpecting the nature and quantity of the diſcharge. In a little time, I was convinced that the flooding was conſiderable, particularly in time of pain; I took the earlieſt opportunity to examine my patient by the touch, and found the vagina filled with what I imagined to be coagulated blood. The os uteri low down, lax, and dilated about the ſize of half a crown. After this examination, the hæmorrhage nearly ceaſed, but ſoon returning to an alarming degree, I determined to ſeek for the fœtus and deliver. On introducing my hand for that purpoſe, the uterus forcibly contracted, and filled my hand with what I thought clots of blood: at that inſtant my patient was ſeized (as ſhe called it) with a moſt violent cramp in her belly, and was attacked with an univerſal rigor; under theſe circumſtances, and ſenſible that the flooding had ceaſed, I deſiſted from purſuing my intention of delivering, and adminiſtered wine and ſuch other remedies as the occaſion ſeemed immediately to require. At this time, the rigor and coldneſs were like the paroxyſm of a ſevere ague, but the pulſe, though quick, was not alarmingly low. When my patient had regained her natural warmth, I was ſolicitous to aſcertain the ſtate of the uterine diſcharge; I found hardly any, I therefore employed myſelf in clearing the bed from the collected coagula; when, to my ſurpriſe, I nearly filled a baſon with innumerable hydatids, of various ſizes, from a large Portugal grape to a ſmall pin's head. The whole, ſo cleared, meaſured three pints and four ounces. The lady recovered her accuſtomed ſhare of health, as patients uſually do after violent floodings, and I believe ſhe has not proved pregnant ſince. She has had had many children. After the birth of the two laſt, her life was in imminent danger, from a flooding that enſued ſoon after the expulſion of the placenta :—I ſay the expulſion of the placenta, becauſe it came away each time with ſcarce any aſſiſtance beyond the natural pains.

